# The role of tobacco use on dental care and oral disease severity within community dental clinics in Japan

**DOI:** 10.1186/1617-9625-11-13

**Published:** 2013-06-21

**Authors:** Miki Ojima, Takashi Hanioka, Kaoru Shimada, Satoru Haresaku, Mito Yamamoto, Keiko Tanaka

**Affiliations:** 1 Department of Preventive Dentistry, Graduate School of Dentistry, Osaka University, Suita, Osaka 565-0871, Japan; 2 Section of Oral Public Health, Fukuoka Dental College, Fukuoka 814-0193, Japan; 3 Section of Medical Statistics, Department of Preventive and Public Health Dentistry, Fukuoka Dental College, Fukuoka 814-0193, Japan; 4 Department of Preventive Medicine and Public Health, Faculty of Medicine, Fukuoka University, Fukuoka 814-0180, Japan

**Keywords:** Dental care, Dental caries, Dental clinic, Periodontal disease, Prosthetic treatment, Smoking

## Abstract

**Background:**

To examine facilitators of dental smoking intervention practices in Japan, where smokeless tobacco is rarely used, we evaluated the characteristics of dental care for smokers.

**Methods:**

Community dentists volunteered to record the treated disease or encounter with patients that was principally responsible for their dental care on the survey day. Patients were classified into groups receiving gingival/periodontal treatment (GPT), caries/endodontic treatment (CET), prosthetic treatment (PRT), periodical check-up/orthodontic treatment (POT), or other encounters/treatments. Potential effect of dentist clustering was adjusted by incorporating the complex survey design into the analysis.

**Results:**

Data of 2835 current smokers (CS) and 6850 non-smokers (NS) from 753 clinics were analysed. Distribution of treatments significantly differed between CS and NS (P = 0.001). In ad hoc multiple comparisons for each treatment, CS were significantly higher than NS for CET (47.1% vs. 43.6%, P = 0.002), and lower for POT (1.6% vs. 2.7%, P = 0.001), whereas GPT and PRT proportions were equivalent by smoking. When stage of disease progression was compared in the GPT subpopulation, CS were more likely received treatment for advanced stage disease than NS in the age groups of 40–59 years (24.9% vs. 15.3%, P = 0.001) and more than 60 years (40.8% vs. 22.1%, P < 0.001). However, the difference was less apparent in the entire population (9.7% vs. 6.0%), and CS were not predominant among patients receiving GPT for advanced stage disease (37.6%).

**Conclusions:**

The association of smoking with type of dental care of CET and GPT severity would warrant the need for dental professionals to engage their patients smoking within clinical practice. The detrimental effects of smoking in dental care for smokers, as evidenced by the distribution of treatment and encounter and stage of treated disease, may not be clearly realized by dental professionals, unless the smoking status of all patients is identified.

## Background

Tobacco use is a modifiable risk behaviour in oral disease development. Dental professionals are able to reduce the oral and overall disease burden related to tobacco by counselling their patients. To date, however, tobacco intervention practices by community dental professionals have been limited, and dental professionals may have not fully embraced opportunities for tobacco use interventions [1], although tobacco use cessation clinical activities have been developed in both dental and dental hygiene programs [2].

Within undergraduate dental education for tobacco use interventions, the most important facilitator is evidence of the effects of tobacco use on oral health [3], while screening guides an important visual tool of the effects of tobacco use on oral health, particularly mucosal lesions [4]. These activities have been mainly conducted in the United States, where dental professionals may have been aware of the detrimental effects of smokeless tobacco in the oral cavity in their daily practice [5]. However, they are now expanding globally based on evidence of the detrimental effects of smokeless tobacco in the United States and European countries [6,7] and in Central Asia [8,9].

Numerous epidemiological studies have shown the detrimental effects of smoking on oral health. The Surgeon General Report in 2004 summarized evidence regarding causality of the association between smoking and oral health [10]. Evidence is sufficient to infer a causal relationship with smoking for cancers of the oral cavity and pharynx, and periodontitis. Evidence is suggestive but not sufficient to infer a causal relationship between smoking and root-surface caries and between maternal smoking and oral clefts. Furthermore, smokers have more missing teeth than non-smokers [11]. Dentist awareness of evidence for the detrimental effects of smoking during actual dental practice may promote smoking cessation activity.

Characterization of dental care for smokers is likely an important factor in determining whether community dentists can actively participate in smoking cessation interventions in countries in which smokeless tobacco has rarely been used. Because smokers appear to experience dental disease more frequently than non-smokers, smokers were more likely to have more perceived dental needs compared with non-smokers [12]. However, smokers are less likely to report dental visits than non-smokers [13]. These contradictory findings hinder the evaluation of the characteristics of dental care for smokers in community dental clinics. To our knowledge, no study has compared dental care between smokers and non-smokers in community dental clinics. The aim of present study was to clarify the characteristic of dental care of smokers in community dental clinics Japan.

## Methods

### Study design

To examine the characteristics of dental care for smokers in community dental clinics, 1022 dentists were randomly selected from among general practitioner members of the Japan Dental Association. Each dentist was notified by mail prior to sending the survey. Dentists were provided with a survey form for data collection, a cover letter outlining instructions on how to complete the survey, and an addressed return envelope for returning the completed survey. The patient survey was conducted by each dentist on a designated day in February 2008. Non-respondents were asked to complete an identical survey in May or July 2008. Patients aged ≥20 years who received dental care from the dentist on the designated day were informed of the study protocol and asked by their dentist about their willingness to participate. The study protocol was approved by the Ethical Committee of Fukuoka Dental College (Ethics Approval no. 115).

### Treated dental disease or patient encounters

Dental care can be determined by examining treated diseases and patient encounters. Community dentists were asked to report on treated diseases and patient encounters on the survey day using a questionnaire that was identical to the Dental Clinic Questionnaire of the Patient Survey conducted in October 2005 by the Ministry of Health, Labour and Welfare of Japan [14], except for the items regarding smoking status. The treated diseases and encounters were presented as specific codes that are commonly used to describe dental diseases in the universal health insurance system in Japan.

Dentists were instructed to select one treated disease or encounter per patient that was principally responsible for the specific dental care on the survey day from a list consisting of 15 items in the questionnaire (Table 1). For example, periodontal disease progression was presented as P_1_, P_2_ and P_3_, representing mild, moderate and severe periodontal disease, respectively. These codes were determined in accordance with the degree of alveolar bone resorption, probing pocket depth and tooth mobility, and are routinely used by community dentists in the universal health insurance system of Japan. Since subgroup analysis requires a sufficient number of patients in each category, the 15 original items were classified into 5 category groups to allow the estimation of characteristics of dental care for smokers, namely gingival/periodontal treatment (GPT), caries/endodontic treatment (CET), prosthetic treatment (PRT), periodical check-up/orthodontic treatment (POT), and other encounters/treatments (OET).

**Table 1:** Summarization of items for treated diseases and encounters

**Category used for analysis**		**Treated disease and encounter recorded in the questionnaire **^**a)**^
Gum/periodontal treatment (GPT)	Early stage ^b)^	Gingivitis
		Mild to moderate periodontal disease ^c)^
	Advanced stage ^b)^	Severe periodontal disease ^c)^
Caries/endodontic treatment (CET)	Early stage ^b)^	Dental caries
	Advanced stage ^b)^	Inflammation of dental pulp
		Apical periodontitis
		Periapical abscess and radicular cyst
Prosthetic treatment (PRT)		Prosthetic treatment
Periodical check-up/orthodontic treatment (POT)		Dental examination ^d)^
		Orthodontic treatment
Other encounters/treatments (OET)		Other periodontal diseases ^e)^
		Other disorders of teeth and supporting structures
		Other diseases of the oral region, salivary glands and jaws
		Stomatitis and related lesions
		Dental injuries

^a)^ Items were recorded in the questionnaire on the survey day into five categories for analysis.

^b)^ This category was used for analysis to compare the distribution of levels of treated diseases within the GPT and CET subpopulations.

^c)^ Periodontal disease progression was presented as P_1_, P_2_ and P_3_ in the questionnaire, representing mild, moderate and severe periodontal disease, respectively. These codes were adopted according to levels of alveolar bone resorption, probing pocket depth and tooth mobility, and are used by general dentists in the universal health insurance system of Japan.

^d)^ This category is mainly applicable to health check-ups.

^e)^ This category is mainly applicable to pericoronitis and gingival abscess.

### Determination of smoking status

The smoking status of patients was determined using a self-administered questionnaire and defined as follows: 1) ‘current smoker’, an individual who currently smokes and has smoked >100 cigarettes in his/her lifetime; 2) ‘former smoker’, an individual who has previously smoked >100 cigarettes, but does not currently smoke; and 3) ‘non-smoker’, an individual who has never smoked or who has smoked ≤100 cigarettes. The use of oral/smokeless tobacco was not assessed in this study because this type of tobacco is rarely used or sold in Japan, and was accordingly expected to have little impact on dental care among our study patients.

### Statistical analyses

The sampling strategy of dental patients consisted of two steps: selection of community dentists, and survey by each dentist of his or her individual patients. Under this complex survey design, community dentists were established as the primary sampling unit of the stratum in the analyses. Former smokers were excluded from analysis, since interpretation of the potential effects of former exposure to tobacco smoking would be difficult.

**Figure 1:**
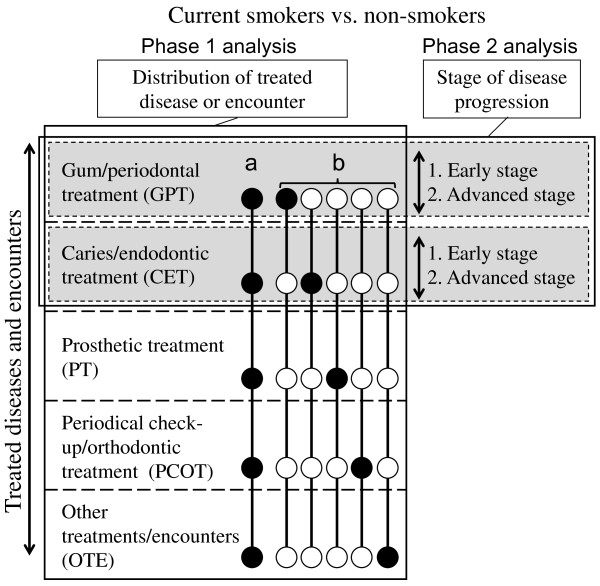
**Analyses of the differences in dental care between current smokers and non-smokers.** Analyses in the present study were conducted in two phases, namely difference in the distribution of treated diseases or encounters (phase 1 analysis), and stage of disease progression (phase 2 analysis). When a significant difference was detected in overall distribution (**a**), five additional 2 × 2 contingency tables were prepared to estimate which group of treated diseases and encounters contributed to the difference in distribution by smoking status (**b**).

Analyses were conducted in two phases (Figure 1). Distribution of the five groups of treated disease and encounters was compared for smoking status by constructing a 5 × 2 contingency table using the chi-square test (Phase 1 analysis). When a significant difference was detected, five 2 × 2 contingency tables were constructed to test which group(s) of treated disease and encounter significantly contributed to the difference using the chi-square test. For this analysis, the significance level was shared at P < 0.01 by the Bonferroni correction, since comparison was repeated five times.

In accordance with the level of progression of treated disease, the GPT and CET patients were further divided into two subgroups during the phase 2 analysis (Table 1). The distribution of smoking status in the GPT and CET subpopulations was tested according to progression of the treated disease (Figure 1). Logistic regression models were constructed for analysis of the GPT and CET subpopulations. In the GPT model, dependent variables were defined in a binary fashion, namely patients treated for early stage disease (gingivitis and mild and moderate periodontal diseases) = 0, and those treated for advanced stage disease (severe periodontal disease) = 1; and in the CET model, patients who were treated for early stage disease (dental caries) = 0, and those treated for advanced stage disease (inflammation of dental pulp, apical periodontitis, periapical abscess, and radicular cyst) = 1. When a significant interaction of smoking with gender or age group (20–39 years, 40–59 years and ≥60 years) was detected, the effect of smoking on the stage of treated disease was examined by constructing models stratified by gender/age groups. Adjusted odds ratios (ORs) and 95% confidence intervals (95% CI) were calculated according to smoking status and the stage of treated disease by adjusting for gender/age groups.

The potential effect of dentist clustering was adjusted for using statistical software (IBM SPSS Complex Samples 20.0, IBM Corp., New York, NY, USA). Significance level was set at P < 0.05, except for the tests of multiple comparisons (P < 0.01).

## Results

Of the 1,022 dentists that were mailed, 753 (73.7%) responded to the survey. These dentists provided records for 14,187 patients, while those of 2,912 were excluded because of incomplete information. Smoking rate was measured at 25.1% (2,835/11,275). Former smokers (n = 1,590) were not included in the analyses. Finally, the data of 2,835 current smokers and 6,850 non-smokers were used to evaluate characteristics of dental care for smokers vs. non-smokers in community dental settings in Japan (Table 2).

**Table 2:** Number of non-smokers and current smokers by gender and age groups

**Factor and category**	**Number of patients**		
	**Non-smokers**	**Current smokers**	**Total**
Gender			
Male	1621	1944	3565
Female	5229	891	6120
Age group (years)			
20–39	1425	889	2314
40–59	2055	1122	3177
≥60	3370	824	4194
Total	6850	2835	9685

Former smokers (n = 1590) were not included in the analysis.

Table 3, depicts the distribution of treated diseases and encounters by smoking status in the phase 1 analysis. CET accounted for nearly half of treatments and encounters (44.6%), followed by GPT (28.4%) and PRT (19.9%). The overall distribution of the five groups significantly differed by smoking (P = 0.001). The ad hoc test for each group showed significant difference in CET (P = 0.002) and POT (P = 0.001) by smoking, with current smokers more likely received CET than non-smokers and less likely to receive POT, though the differences were relatively small (47.1% vs. 43.6% and 1.6% vs. 2.7%, respectively). In contrast, proportions of GPT, PRT and OET was almost equivalent between portions of GPT, PRT and OET was almost equivalent between non-smokers and current smokers (28.8% vs. 27.7%, 20.3% vs. 19.0%, and 4.6% vs. 4.7%, respectively).

**Table 3:** Distribution of treated diseases and encounters by smoking status

**Group according to treated disease and encounter**	**Total % (n)**	**Smoking status**		**P-value**	
		**Non-smokers % (n)**	**Current smokers % (n)**	**All groups**^**a)**^	**Per group**^**b)**^
Gum/periodontal treatment (GPT)	28.4 (2755)	28.8 (1971)	27.7 (784)	0.001	0.299
Caries/endodontic treatment (CET)	44.6 (4324)	43.6 (2988)	47.1 (1336)		0.002
Prosthetic treatment (PRT)	19.9 (1929)	20.3 (1389)	19.0 (540)		0.155
Periodical check-up/ orthodontic treatment (POT)	2.4 (230)	2.7 (187)	1.6 (43)		0.001
Other encounters/treatments (OET)	4.6 (447)	4.6 (315)	4.7 (132)		0.902
Total	100 (9685)	100 (6850)	100 (2835)		

^a)^ Chi-square test for all groups.

^b)^ Chi-square test for each group. Five 2 × 2 contingency tables were prepared to test differences in distribution by smoking. Since comparison was repeated five times, significance level in this series was adjusted by Bonferroni correction, made by multiplying each p-value by 5. For convenience of interpretation, significance level was retained at P < 0.05 throughout this table.

In phase 2 of the analysis for the GPT subpopulation, the participants age was identified as a significant factor (P = 0.005). Ad hoc analyses were then performed in the models stratified by age, at 20–39 years, 40–59 years, and more than 60 years (Table 4), while the number of patients increased with age (n = 489, 958, and 1,308, respectively). While the proportion of advanced stage disease in current smokers and non-smokers (4.1% vs. 4.4%) was similar in the younger age group, current smokers in the older two groups were more likely to receive treatment for advanced stage disease than non-smokers (24.9% vs. 15.3% and 40.8% vs. 22.1%). These differences were significant, with ORs adjusted for gender in the two older age groups of 1.67 (95% CI: 1.10–2.56, P = 0.001) and 2.25 (95% CI: 1.62–3.11, P <0.001). When the proportion was compared based on the entire patient population aged more than 40 years, GPT for advanced stage disease accounted for 6.0% (324/5,425) of non-smokers and 9.7% (195/1,946) of current smokers. Current smokers were not predominant among patients treated with advanced stage disease, at 48.3% and 31.9% in the 40–59 and older than 60 years age groups, respectively, and 37.6% for all patients aged 40 years or more (data not shown).

**Table 4:** Proportion of patients treated in the advanced stage of gingival/periodontal disease progression

**Age group**^**a)**^**(n) smoking status**	**Stage of treated disease**		**Proportion of advanced stage **^**d)**^**(%)**	**Adjusted odds ratio **^**e)**^**(95% CI)**
	**Early stage **^**b)**^**(n, % )**	**Advanced stage**^**c)**^**(n, % )**		
20-39 years (489)				
Non-smokers	305 (65.2)	14 (66.7)	4.4	1.00 (Reference)
Current smokers	163 (34.8)	7 (33.3)	4.1	0.81 (0.30–2.19)
Total	468 (100)	21 (100)		
40-59 years (958)				
Non-smokers	516 (66.3)	93 (51.7)	15.3	1.00 (Reference)
Current smokers	262 (33.7)	87 (48.3)	24.9	1.67 (1.10–2.56)
Total	778 (100)	180 (100)		
≥60 years (1308)				
Non-smokers	812 (83.8)	231 (68.1)	22.1	1.00 (Reference)
Current smokers	157 (16.2)	108 (31.9)	40.8	2.25 (1.62–3.11)
Total	969 (100)	339 (100)		

^a)^ Because of significant interaction with age group (P = 0.005), logistic regression analysis was performed in three models stratified by age group.

^b)^ Patients treated for gingivitis and mild to moderate periodontal disease.

^c)^ Patients treated for severe periodontal disease.

^d)^ The proportion was compared by smoking status in the subpopulation receiving gingival/periodontal treatment in three age groups.

^e)^ Adjusted for gender.

In the analysis to test the distribution of CET level by smoking status (Table 5), interactions with gender and age groups were not significant. Current smokers were more likely to receive treatment for advanced stage disease than non-smokers (54.1% vs. 49.2%), and the difference was significant, with an OR adjusted for age and gender of 1.39 (95% CI: 1.19–1.62, P < 0.001).

**Table 5:** Proportion of patients treated in the advanced stage of caries/endodontic disease progression

**Smoking status**	**Stage of treated disease**		**Proportion of advanced stage **^**c)**^**(%)**	**Adjusted odds ratio **^**d)**^**(95% CI)**
	**Early stage**^**a)**^**(n)**	**Advanced stage **^**b)**^**(n)**		
Non-smokers	1519	1469	49.2	1.00 (Reference)
Current smokers	613	723	54.1	1.39 (1.19–1.62)

^a)^ Patients treated for dental caries.

^b)^ Patients treated for inflammation of the dental pulp, apical periodontitis, periapical abscess, and radicular cyst.

^c)^ The proportion was compared by smoking status in the subpopulation receiving caries/endodontic treatment.

^d)^ Adjusted for age and gender.

## Discussion

In this study, we compared treated diseases and encounters among individual patients in community dental clinics, and found that dental care for current smokers and non-smokers in this setting differed. Current smokers were significantly higher than non-smokers in distribution for CET, and lower for POT, and more likely received GPT for advanced stage disease than non-smokers in older age groups. Our hypothesis was that community dentists in countries in which smokeless tobacco products are rarely used may not realize the detrimental effects of smoking. Therefore, the principal concept to discuss the results should be focused on the characteristic of dental care provided for current smokers. Consideration should be given to health behaviour, particularly less frequent visits to the dentist, although unmet needs of dental treatment were beyond the scope of the present survey.

Our results showed that current smokers were more likely to receive CET and less likely to receive POT than non-smokers. This trend likely reflects the less effective oral hygiene behaviours of smokers [15] and their less frequent visits to dentists [9], respectively. However, since the differences in distribution between non-smokers vs. current smokers were relatively small, community dentists may not realize the significance of these differences in routine clinical settings.

Unexpectedly, we found that the distribution of GPT and PRT between current smokers and non-smokers was similar. If the effects of smoking on periodontal disease [16] and early tooth loss [17,18] in the Japanese population are directly reflected in dental care, proportions of GPT and PRT in current smokers would be higher than those in non-smokers. Again, the less frequent visits by smokers [13] may be responsible for the similarity in proportions of GPT and PRT between current smokers and non-smokers. Because gingival bleeding is suppressed in smokers [19], smokers who were less likely aware of periodontal disease may less likely to visit dentists than non-smokers. Alternatively, certain cases receiving PRT would be attributable to a severe form of periodontal disease. Community dentists may not realize the detrimental effects of smoking from the distribution of treated diseases.

The characteristics of dental care for smokers due to the detrimental effect of smoking may be reflected within the GPT subpopulation, in which the proportions of treatment for advanced stage disease in the two older age groups were 15.3% vs. 24.9% and 22.1% vs. 40.8% for non-smokers vs. current smokers. Dental professionals may not recognize the detrimental effect of smoking since non-smokers were still dominant among patients receiving GPT for advanced stage disease, at 62.4% of patients aged more than 40 years, albeit that former smokers were excluded from analysis.

The characteristics of GPT in smokers may be less apparent when the proportion is compared among the entire patient population, 6.0% vs. 9.7%. The finding would be more clearly reflected in the practice of periodontists, who primarily treat periodontal patients. Among the 5,879 dentists who responded to the nationwide survey in 2009 (response rate, 59%), the ratio of dentists who reported items regarding smoking history in the medical questionnaire and who routinely asked about smoking status was about 30% for both; and although 90% agreed that dentists should advise their patients to quit smoking, only 20% actually do so [20]. Thus, community dentists may be unaware of the smoking status of their patients, and may not recognize the detrimental effect of smoking during GPT.

The deterioration of untreated dental caries often involves more severe destruction of tooth structure, which may require endodontic-related treatment. Smokers may be more likely to be treated for advanced stages of disease of the tooth structure, but this may also reflect the less frequent visits to dentists made by smokers [13]. However, the frequent need for CET in smokers is in part likely a reflection of the detrimental effects of smoking. The Surgeon General Report in 2004 concluded that evidence to infer the presence or absence of a causal relationship of smoking with coronal dental caries is insufficient [10], and the evidence that is available was based on epidemiological studies which did not address possible confounders, and inconclusive findings of salivary alteration. In other studies that controlled for possible confounders, however, dental caries were significantly and independently associated with smoking [21-25]. A cohort study design may allow the inference of causality [21], and the effects of exposure to tobacco contents on the growth and metabolism of *Streptococcus mutans*[26] and on the expansion of the dental caries area [27] may suggest biological plausibility. A mechanism which acts via this cariogenic microorganism is conceivable, since the effects of tobacco use on periodontal microorganisms [28,29] has been recently added to the list of potential causal pathways [30]. These reports invite further studies to examine whether the potential effect of smoking on tooth structure may influence oral health inequalities.

Although current smokers experience tooth loss more frequently than non-smokers [17,18,31], the proportion of prosthetic treatments between smokers and non-smokers in the present study was equivalent. The significant association of smoking with increased risk of prosthetic dental restorations was independent of sociodemographic characteristics, including education and income [32]. Our findings regarding CET and GPT for advanced stage disease in these subpopulations likely warrants further study regarding the intensity of PRT. Studies of Japanese workers revealed that current smokers were more likely to incur higher dental care costs than never or former smokers [33]. The increased proportion of patients receiving care for advanced stage conditions in the CET and GPT subgroups suggests the need for more intensive treatment in smokers than non-smokers.

Several limitations of our study warrant mention. First, a relatively high proportion of patients (20%) was excluded due to incomplete data. However, the smoking rate among the excluded patients was similar to that of the included patients (24.3% vs. 25.1%; data not shown), suggesting that bias caused by the exclusion of data would be limited with respect to smoking. Second, there is no information about diagnosis and socioeconomic status in the present study. Since we looked into treatments rendered by the questionnaire, it is thus difficult to draw concrete conclusions about the exact diagnosis and prognosis. It is possible that there is significant unmeasured confounding by socioeconomic status. These variables should be included to compare diagnosis and prognosis between current smokers and non-smokers in community dental clinics. Third, the survey was conducted on a single day and may have been influenced by daily variations due to weather conditions or weekly periodicity. Any effect of this single-day sampling on the representativeness of dental patients might have been small, however, given that patient distribution for gender and age was similar to that in a patient survey conducted by the Japanese Ministry of Health, Labour and Welfare in October 2005.

## Conclusions

The association of smoking with type of dental care of CET and GPT severity would warrant the need for dental professionals to engage their patients smoking within clinical practice. The detrimental effects of smoking in dental care for smokers regarding the distribution of treatments and encounters and stage of disease at the time of treatment might not be clearly realized by dental professionals unless smoking status of all patients is identified.

### Consent

Written informed consent by filling out the questionnaire was obtained from the patient for the publication of this report.

## Abbreviations

GPT: Gingival/periodontal treatment; CET: Caries/endodontic treatment; PRT: Prosthetic treatment; POT: Periodical check-up/orthodontic treatment; OET: Other encounters/treatments; OR: Odds ratio.

## Competing interests

The authors declare that they have no competing interests.

## Authors’ contributions

MO analyzed the data and interpreted the results of periodontal treatment. TH organized the survey and prepared the manuscript. KS advised on the statistical analyses. SH interpreted the results of prosthetic treatment. MY conducted a behavioral interpretation of the interaction between dental professionals and smokers. KT interpreted the results of dental caries. All authors approved the final version of the manuscript.

## References

[B1] Hanioka T Ojima M Kawaguchi Y Hirata Y Ogawa H Mochizuki Y Tobacco interventions by dentists and dental hygienists Jpn Dent Sci Rev 2013 49 47 56 10.1016/j.jdsr.2012.11.005

[B2] Weaver RG Whittaker L Valachovic RW Broom A Tobacco control and prevention effort in dental education J Dent Educ 2002 66 426 429 11936234

[B3] Barker GJ Williams KB Tobacco use cessation activities in U.S. dental and dental hygiene student clinics J Dent Educ 1999 63 828 833 10608929

[B4] Mecklenburg RE Christen AG Gerbert B Gift HC Glynn TJ Jones RB Lindsay E Manley MW Severson H Tobacco effects in the mouth. A National Cancer Institute and National Institute of Dental Research guide for health professionals 1994 Bethesda, Md: DHHS, USPHS, NIH, NCI (NIH Publication 94–3330)

[B5] Mecklenburg R Scotts RC Chapter 8. Recommendations for the control of smokeless tobacco Smokeless tobacco or health: an international perspective, Smoking and Tobacco Control Monograph No.2 1992 337 350 http://cancercontrol.cancer.gov/brp/tcrb/monographs/2/m2_8.pdf

[B6] Kallischnigg G Weitkunat R Lee PN Systematic review of the relation between smokeless tobacco and non-neoplastic oral diseases in Europe and the United States BMC Oral Health 2008 8 13 10.1186/1472-6831-8-13 18452601 PMC2390522

[B7] Bunnell A Pettit N Reddout N Sharma K O'Malley S Chino M Kingsley K Analysis of primary risk factors for oral cancer from select US states with increasing rates Tob Induc Dis 2010 8 5 10.1186/1617-9625-8-5 20178620 PMC2837638

[B8] Dangi J Kinnunen TH Zavras AI Challenges in global improvement of oral cancer outcomes: findings from rural Northern India Tob Induc Dis 2012 10 5 10.1186/1617-9625-10-5 22494988 PMC3352301

[B9] Khanna S The interaction between tobacco use and oral health among tribes in central India Tob Induc Dis 2012 10 16 10.1186/1617-9625-10-16 23083419 PMC3484074

[B10] US Department of Health and Human Services Oral cavity and pharyngeal cancers, congenital malformations, infant mortality and child physical and cognitive development, and dental diseases The health consequences of smoking: A report of the Surgeon General 2004 Atlanta, GA: U.S. Department of Health and Human Services, Centers for Disease Control and Prevention, National Center for Chronic Disease Prevention and Health Promotion, Office on Smoking and Health 63 115 577–601, and 732–766

[B11] Hanioka T Ojima M Tanaka K Matsuo K Sato F Tanaka H Causal assessment of smoking and tooth loss: a systematic review of observational studies BMC Publ Health 2011 11 221 10.1186/1471-2458-11-221 PMC3087682 21477320

[B12] Dye BA Morin NM Robison V The relationship between cigarette smoking and perceived dental treatment needs in the United States, 1988–1994 J Am Dent Assoc 2006 137 224 234 16521389 10.14219/jada.archive.2006.0148

[B13] Drilea SK Reid BC Li CH Hyman JJ Manski RJ Dental visits among smoking and nonsmoking US adults in 2000 Am J Health Behav 2005 29 462 471 10.5993/AJHB.29.5.9 16201863

[B14] Health Statistics Office, Statistics and Information Department, Minister’s Secretariat, Ministry of Health, Labour and Welfare, Japan Dental clinic questionnaire of patient survey 2005 Tokyo http://www.mhlw.go.jp/english/database/db-hss/dl/DentalClinicQuestionnaire_sps.pdf

[B15] Andrews JA Severson HH Lichtenstein E Gordon JS Relationship between tobacco use and self-reported oral hygiene habits J Am Dent Assoc 1998 129 313 320 9529806 10.14219/jada.archive.1998.0205

[B16] Ojima M Hanioka T Tanaka K Inoshita E Aoyama H Relationship between smoking status and periodontal conditions: findings from national databases in Japan J Periodontal Res 2006 41 573 579 10.1111/j.1600-0765.2006.00915.x 17076784

[B17] Hanioka T Ojima M Tanaka K Aoyama H Relationship between smoking status and tooth loss: findings from national databases in Japan J Epidemiol 2007 17 125 132 10.2188/jea.17.125 17641448 PMC7058469

[B18] Ojima M Hanioka T Tanaka K Aoyama H Cigarette smoking and tooth loss experience among young adults: a national record linkage study BMC Publ Health 2007 7 313 10.1186/1471-2458-7-313 PMC2186324 17976246

[B19] Dietrich T Bernimoulin JP Glynn RJ The effect of cigarette smoking on gingival bleeding J Periodontol 2004 75 16 22 10.1902/jop.2004.75.1.16 15025212

[B20] Imai H Hayashi K Survey on smoking cessation intervention in dental clinics in Japan Support and promotion of the comprehensive achievement on health promotion plan by tobacco control. A report of the Health and Labour Sciences Research Grants for Clinical Cancer Prevention and Health Services 2010 89 115 Article No. 200925010A

[B21] Bruno-Ambrosius K Swanholm G Twetman S Eating habits, smoking and toothbrushing in relation to dental caries: a 3-year study in Swedish female teenagers Int J Paediatr Dent 2005 15 190 196 10.1111/j.1365-263X.2005.00621.x 15854115

[B22] Bartoloni JA Chao SY Martin GC Caron GA Dental caries risk in the U.S. Air Force J Am Dent Assoc 2006 137 1582 1591 17082285 10.14219/jada.archive.2006.0094

[B23] Becker T Levin L Shochat T Einy S How much does the DMFT index underestimate the need for restorative care? J Dent Educ 2007 71 677 681 17493976

[B24] Aguilar-Zinser V Irigoyen ME Rivera G Maupomé G Sánchez-Pérez L Velázquez C Cigarette smoking and dental caries among professional truck drivers in Mexico Caries Res 2008 42 255 262 10.1159/000135670 18523384

[B25] Campus G Cagetti MG Senna A Blasi G Mascolo A Demarchi P Strohmenger L Does smoking increase risk for caries? a cross-sectional study in an Italian military academy Caries Res 2011 45 40 46 10.1159/000322852 21228593

[B26] Huang R Li M Gregory RL Effect of nicotine on growth and metabolism of *Streptococcus mutans* Eur J Oral Sci 2012 120 319 325 22813222 10.1111/j.1600-0722.2012.00971.x

[B27] Fujinami Y Nakano K Ueda O Ara T Hattori T Kawakami T Wang PL Dental caries area of rat molar expanded by cigarette smoke exposure Caries Res 2011 45 561 567 10.1159/000331926 22067411

[B28] Kumar PS Matthews CR Joshi V de Jager M Aspiras M Tobacco smoking affects bacterial acquisition and colonization in oral biofilms Infect Immun 2011 79 4730 4738 10.1128/IAI.05371-11 21859855 PMC3257914

[B29] Bagaitkar J Daep CA Patel CK Renaud DE Demuth DR Demuth DR Scott DA Tobacco smoke augments *Porphyromonas gingivalis* - *streptococcus gordonii* biofilm formation PLoS One 2011 6 e27386 10.1371/journal.pone.0027386 22110637 PMC3215692

[B30] Ojima M Hanioka T Destructive effects of smoking on molecular and genetic factors of periodontal disease Tob Induc Dis 2010 8 4 10.1186/1617-9625-8-4 20170537 PMC2836317

[B31] Dietrich T Maserejian NN Joshipura KJ Krall EA Garcia RI Tobacco use and incidence of tooth loss among US male health professionals J Dent Res 2007 86 373 377 10.1177/154405910708600414 17384035 PMC2582143

[B32] Zitzmann NU Staehelin K Walls AWG Menghini G Weiger R Zemp Stutz E Changes in oral health over a 10-yr period in Switzerland Eur J Oral Sci 2008 116 52 59 10.1111/j.1600-0722.2007.00512.x 18186732

[B33] Ide R Hoshuyama T Wilson D Takahashi K Higashi T The effects of smoking on dental care utilization and its costs in Japan J Dent Res 2009 88 66 70 10.1177/0022034508327523 19131320

